# Integration of single-cell multi-omics for gene regulatory network inference

**DOI:** 10.1016/j.csbj.2020.06.033

**Published:** 2020-06-29

**Authors:** Xinlin Hu, Yaohua Hu, Fanjie Wu, Ricky Wai Tak Leung, Jing Qin

**Affiliations:** a Shenzhen Key Laboratory of Advanced Machine Learning and Applications, College of Mathematics and Statistics, Shenzhen University, Shenzhen 518060, China; b School of Pharmaceutical Sciences (Shenzhen), Sun Yat-sen University, Shenzhen 518107, China

**Keywords:** Single-cell sequencing, Gene regulatory network inference, Single-cell multi-omics integration

## Abstract

The advancement of single-cell sequencing technology in recent years has provided an opportunity to reconstruct gene regulatory networks (GRNs) with the data from thousands of single cells in one sample. This uncovers regulatory interactions in cells and speeds up the discoveries of regulatory mechanisms in diseases and biological processes. Therefore, more methods have been proposed to reconstruct GRNs using single-cell sequencing data. In this review, we introduce technologies for sequencing single-cell genome, transcriptome, and epigenome. At the same time, we present an overview of current GRN reconstruction strategies utilizing different single-cell sequencing data. Bioinformatics tools were grouped by their input data type and mathematical principles for reader's convenience, and the fundamental mathematics inherent in each group will be discussed. Furthermore, the adaptabilities and limitations of these different methods will also be summarized and compared, with the hope to facilitate researchers recognizing the most suitable tools for them.

## Graphical abstract

**Figure:**
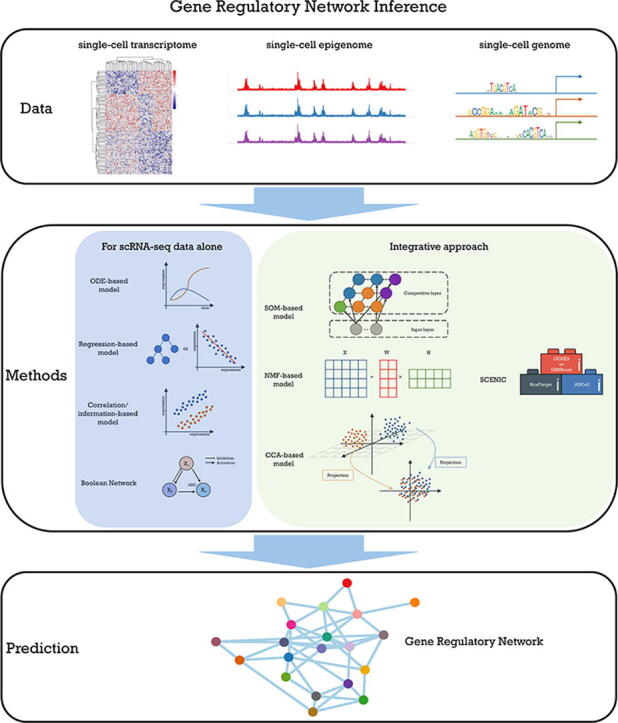

Gene regulatory networks (GRNs), which describe the regulatory connections between transcription factors (TFs) and their target genes, help researchers to investigate the gene regulatory circuits and underlying mechanisms in various diseases and biological processes. A simple model of gene transcriptional regulation includes two key events: (1) an active TF binds to a cis-regulatory element such as a gene promoter; (2) such binding activates/suppresses the expression of the gene, which leads to the increase/decrease of the gene's RNA level. By integrating high-throughput omics data detecting the above two events in genome-wide scale, various powerful methods have been developed for reconstructing GRNs [44], [53], [68], [85]. The recent development of technology makes it possible to sequence the single-cell genome, transcriptome, and epigenome. This provides rich datasets for GRN analyses. However, the inference of GRNs from single-cell sequencing data raises new challenges for method development. One of the main challenges is the underlying phenomenon of missing data. For single-cell transcriptome sequencing, the starting amount of RNAs extracted from single cells are often very low, genes with low or moderate expression are thus being omitted from the followed processing and sequencing steps due to inadequate sensitivities. Moreover, stochastic inherence and cell-to-cell variability of gene expression also result in aggravated noises [37], [54]. For single-cell genome or epigenome sequencing, each DNA molecule in a diploid genome has only one or two opportunities to be sequenced. When only thousands of distinct reads can be detected per cell, it is impossible to cover all sites in the genome. Therefore, single-cell genome and epigenome sequencing suffer data omission even worse than that of transcriptome sequencing. Despite the challenges mentioned, dozens of methods have been developed to predict GRNs from single-cell sequencing data [12], [20], [31], [35], [83]. However, selecting the proper tool according to one's needs is not an easy task for biological/biomedical researchers, as they are usually not very familiar with the mathematical reasoning behind these tools. Thus, understanding the basic principles of the algorithms implemented in these tools and their adaptabilities facilitates researchers making suitable choices according to their needs. In the following sections, we will be introducing, grouping, and discussing current GRN reconstruction strategies. This would also help tool developers to improve their tools by comparing the advantages and disadvantages of different methods. This review focuses on the representative and popular GRN inference approaches which utilize single-cell sequencing data especially on those with multi-omics data integration that can likely improve their performances (Table 1).

**Table 1:** Summary of bioinformatics tools for GRN reconstruction from single-cell sequencing data.

Data	Methods	Name	Reference	Data dimension (cell*gene)	Adaptability
scRNA-seq alone	ODE-based	SCODE	[70]	mESC:456*100 Fibroblast:405*100 hESC:758*100	Reduced computational complexity; assume all cells are on the same trajectory; the linear relationship between change rate of target gene and expression of input is assumed; require expression data with temporal information.
		GRISLI	[5]	Embryonic:373*40 Hescs:758*49	Consider multiple trajectories; assume that each gene is regulated by only a few TFs; expression change rate of target gene and TFs is assumed to be linearly related; require expression data with temporal information.
		InferenceSnapshot	[78]	HSCs:597*18	Directly extract temporal information from single-cell snapshot data; reconstruct more complicated network; limited ODE-based models are considered; the accuracy of the final network may be affected by the initial coarse GRN generated from GENIE3; limit to small-sized GRNs.
	Regression-based	GENIE3	[97]	*E. coli*:907*4297	Not require temporal information; explain more complicated underlying GRNs; fast running when using parallel computation.
		SINCERITIES	[80]	THP-1:960*45	Low computational complexity; parallel computation is available; the relationship between distributional distance of the regulators and target gene is assumed to be linear; require expression data with temporal information.
	Correlation\information-based	LEAP	[90]	Dendritic:564*557	Fast and efficient algorithm; identify more interactions; relationships between all genes are assumed to be linear; require expression data with temporal information.
		PIDC	[18]	MEP:681*87 Embryonic:3934*20 Hematopoietic:442*46	Not require temporal information; consider more complicated information from data; influenced by the choice of data discretization methods and MI estimators; high computational complexity but could be relieved by Julia.
		Scribe	[86]	*C. elegans*:184442*265	Consider more complicated structure of underlying GRN; assume that underlying processes can be described by a first-order Markov process; high computational complexity; require expression data with temporal information.
	Boolean network	SCNS toolkit	[74]	mESC:3934*42	When applied to different stages of cell population, it can be used to reveal the developmental trajectory of the whole organ from the single-cell level, but the increase of gene number will significantly increase the computation, and it is limited in small-size GRNs.
scRNA-seq with genome	Motif	SCENIC	[2]	Mouse brain:3005*151 Human neurons:3083*259 Human brain:466*259	GRN can be reconstructed to identify cell states at the same time, which means that it can be applied to data sets with complex cell states.
scRNA-seq with scATAC-seq	SOM	LinkedSOMs	[52]	Mouse pre-B: 128*12380 (scRNA-seq) + 227*25466 (scATAC-seq)	Provide a framework for integration of different types of data; SOM may spend a long time to converge.
	NMF	Coupled NMF	[30]	mESCs: 463*21973 (scRNA-seq) + 415*23180 (scATAC-seq)	Provide a framework for integration of different types of data; the expression of a subset of genes is assumed to be linearly predicted from the status of chromatin regions; quickly converge but no convergence properties are established.
	CCA	Seurat v3	[91]	Mouse visual cortex: 14249*34617 (scRNA-seq) + 2420*? (scATAC-seq)	Provide a framework for integration of different types of data; the output of this method is an integrated expression matrix that could be used in any single-cell GRN inference method.

## 1 Single-cell sequencing for GRN reconstruction

Different from bulk sequencing that averages signals from a bulk of cells, single-cell sequencing isolates single cells from cell populations and labels DNA molecules derived from every single cell with unique barcodes before next-generation sequencing [25]. Single-cell RNA sequencing (scRNA-seq), the most popular single-cell sequencing technology, sequences RNA molecules in each cell and quantifies their expression levels. It can capture gene expression stochasticity and dynamics while revealing transcriptome-wide cell-to-cell variability at a high resolution [83]. With thousands of genes in hundreds to thousands of single cells being measured by scRNA-seq, TF-gene interactions could be inferred based on the dependency of their expression. Thus, scRNA-seq data becomes one of the major data sources for GRN construction. Single-cell epigenome sequencing is another way to explore the regulatory relationship between TF and gene. Single-cell assay for transposase-accessible chromatin with sequencing (scATAC-seq) [16] detects the chromatin accessibility in single cells. scATAC-seq allows the identification of DNA regulatory elements within accessible genomic DNA regions in single cells. Similarly, single-cell chromatin immunocleavage sequencing (scChIC-seq) profiles histone modifications such as H3K4me3 in single cells, some of which detect DNA regulatory regions during gene regulations, for example, regions associated with transcription activations [57]. Meanwhile other single-cell sequencing techniques such as single-cell reduced representation bisulfite sequencing (scRRBS) [39], single-cell whole-genome bisulfite sequencing (scWGBS) [34], genome-wide CpG island (CGI) methylation sequencing for single cells (scCGI-seq) [41] and single-cell bisulfite sequencing (scBs-seq) [22] were developed for detecting DNA methylation profiles throughout single-cell genomes. With these single-cell epigenome data, GRN could be reconstructed by inferring TFs that bind to the genes with open or active DNA regulatory elements and epigenetic modifications, which indicates potential direct regulations between the TFs and the target genes. In addition, single-cell genome sequencing that detects genomic variations among single cells is a powerful tool to explore genetic heterogeneity and reconstruct cell lineage hierarchies of complex samples, such as tumor tissues. Mutations located at genomic DNA regulatory elements are also an important inducer of disease and affect the underlying gene regulatory network [73], thus the information of genomic variations in single cells is also valuable for GRN reconstruction. Another method screening genetic perturbation pool after clustered regularly interspaced short palindromic repeats (CRISPR)- mediated gene inactivation is called Perturb-seq or CROP-seq, which is very useful for reverse genetics and thus GRN constructions when combined with scRNA-seq [28]. It can also be used to verify inferred GRNs by perturbing selected TFs in the network.

Furthermore, there are techniques able to detect more than one type of single-cell omics profiles simultaneously. For example, single-cell genome and transcriptome sequencing (G&T-seq) [67], gDNA-mRNA sequencing (DR-Seq) [27] and single-cell transcriptogenomics (SCTG) [62] are techniques examining transcriptome and genome sequences in the same single-cell at the same time. Single-cell DNA methylome and transcriptome sequencing (scMT-seq) [49] and (scM&T-seq) [4] are able to detect methylome and transcriptome in parallel to explore the cellular connections between epigenetic variation and transcriptional regulation. Single-nucleus chromatin accessibility and mRNA expression sequencing (SNARE-seq) [19] draws the combined map of chromatin accessibility and mRNA expression in the same cell. Single-cell nucleosome occupancy and methylome sequencing (scNOMe-seq) measures chromatin accessibility and endogenous DNA methylation in single cells [82]. Some technologies can even measure three types of molecules in single cells. For instance, single-cell nucleosome, methylation and transcription sequencing (scNMT-seq) detects chromatin accessibility, DNA methylation and transcriptome profiling in parallel [21]. Single-cell triple omics sequencing (scTrio-seq) [48] combines single-cell genome, methylome and transcriptome. These methods explore how the heterogeneity of genome and epigenome affects transcriptional heterogeneity in the same cells, thus probably enable GRN inference using computational methods originally designed for integrating bulk sequencing of multiple omics [88].

**Fig. 1:**
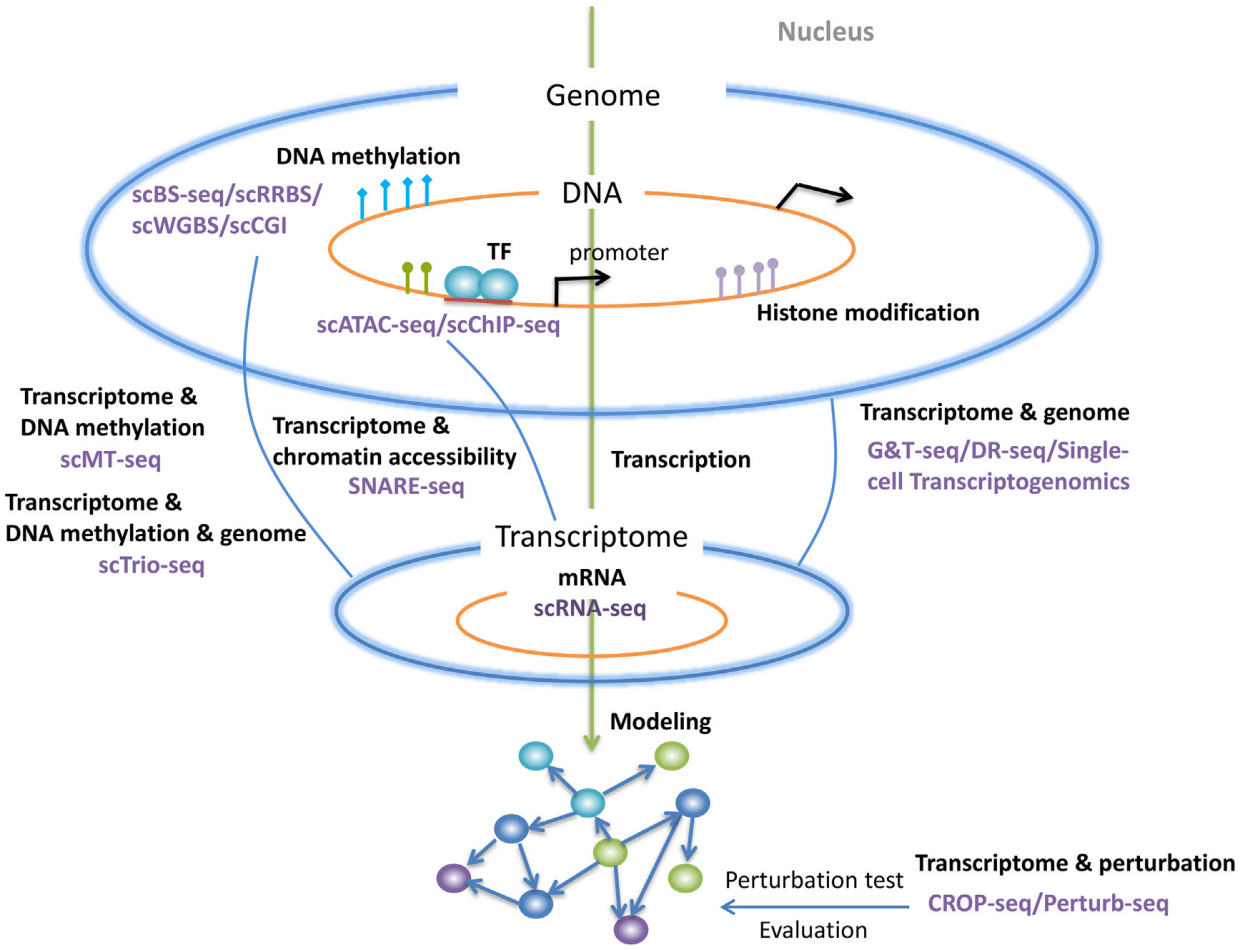
Single-cell sequencing technologies that investigate gene regulatory mechanisms.

**Fig. 2:**
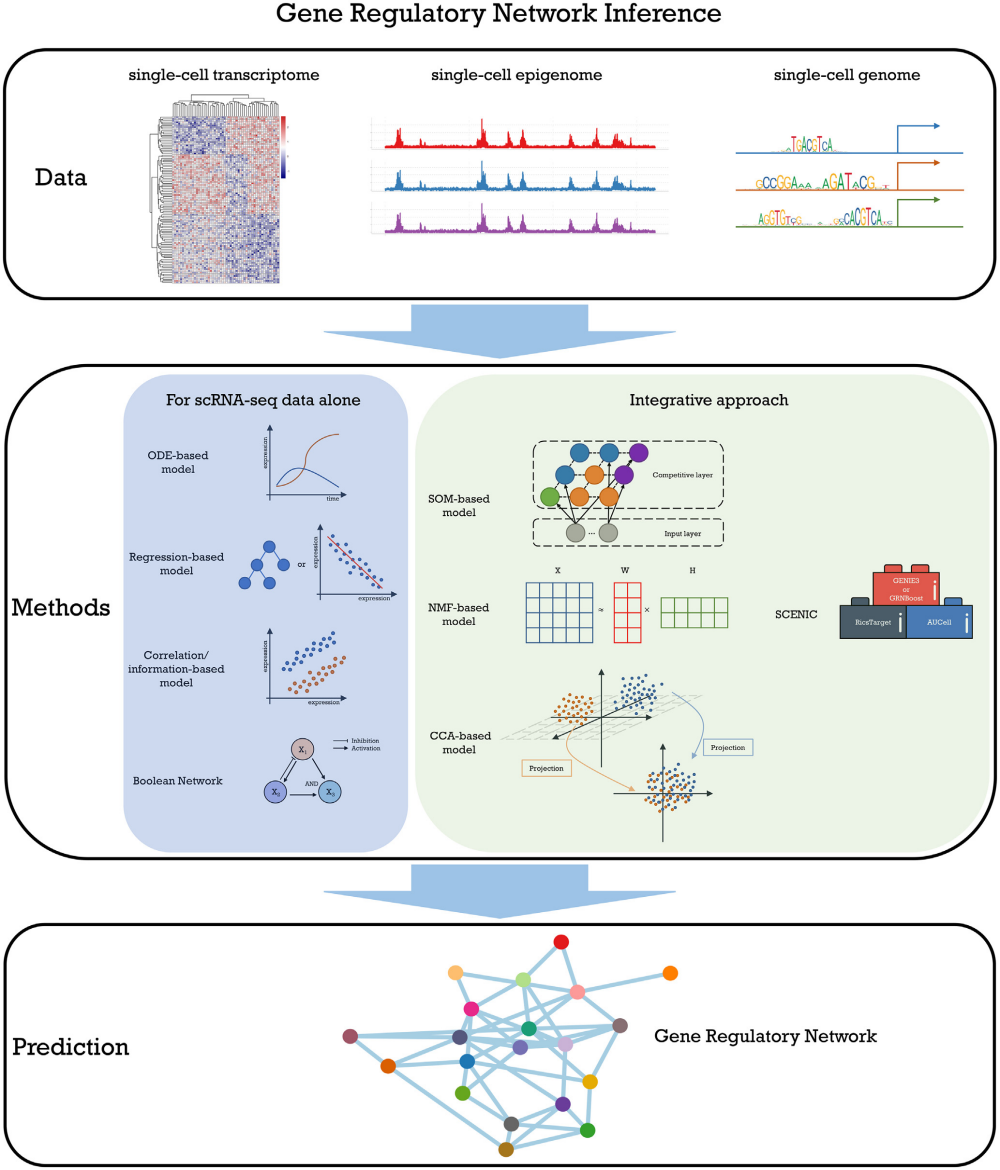
The summary of gene regulatory network inference from single-cell sequencing data.

These single-cell omics and multi-omics technologies give us new opportunities to investigate complex gene regulatory mechanisms in a single-cell resolution (Fig. 1). In short, sequencing data of single-cell genome, transcriptome and epigenome provides distinct information for GRN inference. In the following sections, we will discuss several popular strategies and algorithms that incorporate various single-cell sequencing data to construct GRNs (Fig. 2).

## 2 Methods for scRNA-seq data alone

Tools designed for GRN reconstruction from scRNA-seq data alone have been reviewed and evaluated elsewhere [12], [20], [83]. The performance of these tools was compared using simulated and real scRNA-seq data, and results in these studies revealed that there is no one method well accepted to be the best. This may be because different methods are suitable for different types and sources of data. Moreover, in these reviews, the mathematical concepts and basic algorithms implicit in these tools were not discussed in depth. In this section, we introduce four major categories of popular algorithms for inferring GRNs from scRNA-seq data alone: (1) the ordinary differential equation (ODE)-based model, (2) the regression-based model, (3) the correlation/information-based mode and (4) the Boolean network. For each group, the mathematical principle of the algorithm and the representative tools are described to bridge the knowledge gap between method developers and biological/biomedical researchers.

There are two types of scRNA-seq data – with and without temporal information. In a biological process, condition or experiment, cells can be collected from tissues or cell cultures. These cells could be in a process of change or in a steady state. For instance, cells might undergo differentiation, drug treatments, environmental changes, etc., and transit from one condition to another. In these processes, single-cell snapshot data can be obtained by collecting cells at a certain time point. Although each single cell represents a static state at this single time point, cells may have different stochastic behaviors during the same process [32], some sort of temporal information is still retained in this snapshot of cells. Such temporal information, called pseudo-time, can be inferred by the cell trajectory analysis [89], [93]. Based on scRNA-seq data, cells could be ordered along the trajectory of the cell transition process [38], which represents the pseudo-time series. When cells are collected from tissues without any treatment or cells under pooled CRISPR screening, these cells are in a relatively static state or in a large number of independent processes. Cell populations in these samples do not show temporal relationships as those mentioned above. Therefore, when choosing the method/tool to reconstruct a GRN, we need to first determine whether there is temporal information in the single-cell sample, as some methods are designed specifically to work with temporal information, and others are more suitable for those without. While, there are also some methods can analyze both types of data.

### 2.1 ODE-based model

Provided with expression data with temporal information, ODE has been applied to describe expression dynamics and infer GRNs, which is generally formulated as

(1) $$\begin{array}{c} \frac{\mathrm{dy}}{\mathrm{dt}}=f\left(x\right), \end{array}$$

where x and y represent the expression data of TFs and a target, respectively, and both are time series related to time t. The task is to find the function $f\left(x\right)$ and describe the expression change rate of target y, which also depicts how target y is regulated by TFs x.

Assumed that the expression change rate of target y linearly depends on the expression of TFs x, equation (1) is reduced to a simple linear ODE:

(2) $$\begin{array}{c} \frac{\mathrm{dy}}{\mathrm{dt}}=a_{1}x_{1}+a_{2}x_{2}+\cdots a_{n}x_{n}. \end{array}$$

If parameters $a_{1},a_{2},\cdots ,a_{n}$ and the initial values of t and y in equation (2) are provided, equation (2) can be solved by integration. However, the parameters are usually unknown in practice. Hence, the major task is to find the parameters $a_{1}^{*},a_{2}^{*},\cdots ,a_{n}^{*}$ for equation (2) such that the error between estimation $y(a_{1}^{*},a_{2}^{*},\cdots ,a_{n}^{*})$ and observation $\hat{y}$ is minimal [6]. These parameters are also able to imply the regulatory relationships between the target and TF, whose observed expression data are $x_{1},x_{2},\cdots ,x_{n}$. Several algorithms for solving this problem have been investigated by using least squares [63], [107], two-stage methods [45], and so on [64], [104].

#### 2.1.1 SCODE

SCODE is a bioinformatics tool designed for scRNA-seq data by using the linear ODEs with pseudo-time series to describe expression dynamics and infer GRNs [70]. Two important assumptions are made in the SCODE: (1) all cells are on the same trajectory, that is, all cells are differentiating into the same cell type, and (2) the expression change rate of each TF linearly depends on expression profiles of themselves. Thus, the expression dynamics of TFs can be described for all differentiating cells along the pseudo-time series by using the linear ODEs:

(3) $$\begin{array}{c} \frac{dx_{c}}{\mathrm{dt}}=Ax_{c}, \end{array}$$

where $x_{c}:={\left[x_{1},x_{2},\cdots ,x_{T}\right]}_{c}^{⊤}$ denotes the expression of T TFs in cell c at time $t_{c}$, and the square matrix A represents the regulatory network among TFs. More precisely, the ODE (3) for each element $x_{i}$ in vector $x_{c}$ can be reformulated in the form of equation (2):

$$\frac{dx_{i}}{\mathrm{dt}}=A_{i\cdot }x_{c}=A_{i1}x_{1}+A_{i2}x_{2}+\cdots A_{\mathrm{iT}}x_{T},$$

where $dx_{i}/dt$ represents the expression change rate of the ith TF. The task of the ODE-based model is to estimate the matrix A such that the expression change rate of the ith TF at the time $t_{c}$ can be approximately described by all TFs' expression levels.

A major challenge of the ODE-based models is the expensive computational complexity caused by the high dimensionality of samples and genes. To reduce the computational complexity, SCODE alternatively solves an ODE with low-dimensional data by assuming that the high-dimensional data can be linearly expressed in a low-dimensional subspace [70]. In details, suppose that $x_{c}$ can be expressed as a linear regression of a low-dimensional subspace

(4) $$\begin{array}{c} x_{c}=Wz_{c}, \end{array}$$

where $W\in R^{T\times D}$ with D≪T, and $z_{c}$ obeys an ODE

(5) $$\begin{array}{c} \frac{dz_{c}}{\mathrm{dt}}=Bz_{c}. \end{array}$$

Then the equation (3) is reduced to

$$\frac{dx_{c}}{\mathrm{dt}}=WBW^{+}x_{c},$$

where $W^{+}$ denotes the pseudo-inverse matrix of W, and thus, A can be generated by

$$A=WBW^{+}.$$

Solving the ODE (5) in a low-dimensional subspace instead of the ODE (3), the SCODE algorithm significantly reduces the computational complexity and consumes much less running time than the traditional ODE (3). Thus, this method is capable of dealing with large networks, for instance, a network with 5000 genes [83]. However, the linear relationship in ODE might be too simple to describe the regulatory relationships between TFs. In addition, SCODE cannot directly infer GRN from single-cell expression data without temporal information [70]. For example, a tissue sample containing various cell types going through different biological processes is not suitable to be analyzed by this method.

#### 2.1.2 GRISLI

GRISLI is another bioinformatics tool for single-cell pseudo-time-series data based on linear ODE [5], where the expression dynamics are modeled by ODE (3) as in SCODE. While, different from SCODE, GRISLI designs a fast algorithm via solving a linear regression with a response as $dx_{c}/dt$ in ODE (3) instead of integrating the ODE. The inferred GRN is assumed to be sparse, that is, most of elements in matrix A are zero, due to the biological assumption that each gene is regulated by only a few TFs.

Breaking the assumption in SCODE that all cells are in the same trajectory, GRISLI believes that different cells could evolve on different trajectories and focuses on those cells whose trajectories are close to each other. First, the expression change rate, also described as velocity, between cell c and cell e at two close pseudo-time points $t_{c}$ and $t_{e}$ is estimated by

$${\hat{v}}_{c,e}=\frac{x_{c}-x_{e}}{t_{c}-t_{e}}.$$

Considering that some data points might live in the past ($t<t_{c}$) or the future ($t>t_{c}$) of a given data point ${(x}_{c},t_{c})$, the final estimator of velocity ${\hat{v}}_{c}$ of cell c is defined as a weighted average of all velocities between cell c and those cells closed to it, which is written as

$${\hat{v}}_{c}=\frac{1}{2}\frac{\sum_{e|t_{e}>t_{c}}K\left(x_{e},t_{e},x_{c},t_{c}\right){\hat{v}}_{c,e}}{\sum_{e|t_{e}>t_{c}}K\left(x_{e},t_{e},x_{c},t_{c}\right)}+\frac{1}{2}\frac{\sum_{e|t_{e}<t_{c}}K\left(x_{e},t_{e},x_{c},t_{c}\right){\hat{v}}_{c,e}}{\sum_{e|t_{e}<t_{c}}K\left(x_{e},t_{e},x_{c},t_{c}\right)},$$

where the spatio-temporal kernel $K\left(x_{e},t_{e},x_{c},t_{c}\right)$ measures the significance of a point to the velocity estimation. The velocity matrix $\hat{V}:=\left[{\hat{v}}_{1},{\hat{v}}_{2},\cdots ,{\hat{v}}_{C}\right]\in R^{G\times C}$ is then estimated with corresponding expression data $X:=\left[x_{1},x_{2},\cdots ,x_{C}\right]\in R^{G\times C}$, where G and C are the numbers of genes and cells, respectively.

The following procedures are repeated to obtain the frequency of nonzero elements in the estimated matrix $\hat{A}$: (1) data $\left(\overset{\;}{\overset{\sim }{X}},\overset{\;}{\overset{\sim }{V}}\right)$ are generated by randomly subsampling and multiplying each row i of X by a random number; see section Methods in [5] for details; (2) the Lasso regression [94],

$$\underset{A_{j\cdot }\in {\mathbb{R}}^{G}}{\min}{\left\|{\overset{\;}{\overset{\sim }{V}}}_{j\cdot }-A_{j\cdot }\overset{\;}{\overset{\sim }{X}}\right\|}^{2}+\lambda {\left\|A_{j\cdot }\right\|}_{1},$$

is then solved for each row j to obtain a sparse matrix $\hat{A}$, where ‖·‖ and ${\left\|\cdot \right\|}_{1}$ denote sum of squared values and absolute values, respectively, of all elements in the vector. The penalty parameter λ is set to satisfy the required number of nonzero entries in the row vector of A. After repetition of above procedures, the final GRN can be inferred based on the area score [43] or the original stability selection score [72] calculated from the frequency of occurred regulatory links (nonzero elements in the estimated matrix $\hat{A}$).

As GRISLI describes expression dynamics by linear ODE as SCODE does, the problem is transformed as a sparse regression under the assumption that inferred GRN is sparse. GRISLI is more efficient to estimate the matrix A via solving a convex optimization problem rather than integrating the ODE, and more genes (but less than 1000 genes) can be considered in practice [83]. Moreover, it allows cells to be on different trajectories, which suits for more realistic and general cases. For example, cells may differentiate into two types of cells simultaneously. However, the same as SCODE, GRISLI cannot reconstruct the GRN directly from scRNA-seq data without temporal information.

#### 2.1.3 InferenceSnapshot

InferenceSnapshot is a modular skeleton to extract the temporal information and capture gene expression dynamics directly from scRNA-seq snapshot data [78]. By combining the diffusion map algorithm for dimensionality reduction [23] and ad hoc algorithm for clustering, the low-dimensional data can be obtained and separated into several branches with different cellular processes. Pseudo-time series is generated by using the Wanderlust algorithm [7] to order single cells along discrete paths that represent pseudo-time variables. Two types of ODE-based models are used to describe the interactions between M TFs $x_{i}\left(i=1,\cdots ,M\right)$ and target gene y, representing AND and OR logic gates when combining regulatory effects of TFs, which are respectively formulated as

$$\frac{\mathrm{dy}}{\mathrm{dt}}=\alpha \prod_{m=1}^{M}f_{m}\left(x_{m}\left(t\right),\theta_{m}\right)-\mu y,$$

$$\frac{\mathrm{dy}}{\mathrm{dt}}=\alpha \sum_{m=1}^{M}f_{m}\left(x_{m}\left(t\right),\theta_{m}\right)-\mu y,$$

where α and μ denote the production rate and decay rate of target gene expression, respectively, and

$$f\left(x\left(t\right);\kappa ,b\right):=\left\{\begin{array}{c} \frac{x^{b}}{x^{b}+\kappa^{b}},\;\mathrm{if}\;x\;\mathrm{is}\;\mathrm{activating},\\ \frac{\kappa^{b}}{x^{b}+\kappa^{b}},\;\mathrm{if}\;x\;\mathrm{is}\;\mathrm{inhibiting}. \end{array}\right)$$

Markov chain Monte Carlo based method is used to estimate the parameters in ODE-based models mentioned above. In the model selection process, a coarse GRN is generated by GENIE3 [97] as prior knowledge, and Bayes' factors are computed to select the ODE model from Bayesian model comparison through thermodynamic integration [17].

InferenceSnapshot makes it possible to extract pseudo-time series from snapshot data directly and allows the analysis of data with multiple trajectories. Using the nonlinear function and different logic to combine regulatory effects of multiple TFs, InferenceSnapshot can be used to describe more complicated networks and nonlinear expression relationships, but difficult to be scaled up to large networks due to high computational complexity of ODE and Bayesian models (e.g., a network with 18 genes is considered in the original study) [78]. Moreover, the accuracy of the final network may be affected by the initial coarse GRN generated from GENIE3.

### 2.2 Regression-based model

Different from the ODE that considers expression change rate, the regression-based model is built on the assumption that the expression of a target gene can be predicted by the expression of TFs regulating it. Regression is one of the most commonly used methods to search for a suitable prediction function f to characterize the underlying networks. For example, if the expression data of gene y can be predicted by the expression data of TFs x, then those TFs jointly regulates gene y. Hence, the regression model is written as

(6) $$\begin{array}{c} y=f\left(x\right)+\varepsilon , \end{array}$$

where ε denotes the noise in data. The function f in the regression model can be either linear or non-linear, depending on the assumption of the structure of the target network.

A significant benefit of the regression model is that it is simple to understand and convenient to apply to the complicated biological system [83], [84]. When the prediction function f is provided according to the biological process or data observation, ordinary least squares is a popular method used to solve the regression model (6) to estimate the coefficients involved in f, which aims to minimize the sum of squared errors between the prediction and the true data, that is,

(7) $$\begin{array}{c} \min{\|f\left(x\right)-y\|}^{2}. \end{array}$$

The most common form of regression is linear regression and the associated linear least squares method. Furthermore, the structure of the GRNs can be characterized by adding an associated penalty function p in the regression model to improve the accuracy and stability of prediction, that is,

$$\min{\|f\left(x\right)-y\|}^{2}+\lambda p\left(x\right).$$

For example, ridge regression uses the $l_{2}$ penalty (i.e., $p\left(x\right)={\left\|x\right\|}^{2}$) to measure the magnitude of coefficients [46]; Lasso regression employs the $l_{1}$ penalty (i.e., $p\left(x\right)={\left\|x\right\|}_{1}$) to induce the sparsity of variables [94]. Moreover, the low-order penalized Lasso [84] and fused Lasso have been used in GRN inference [79].

Another important benefit of the regression model is the exclusive development of optimization algorithms. Several popular and efficient numerical algorithms have been proposed to solve the least squares problem (7) and the ridge regression problem such as gradient descent methods, Newton-type methods and Levenberg-Marquardt method [9], [51], [76]. Many state-of-the-art algorithms have been designed and applied to solve the Lasso-type regression models such as proximal/projected gradient methods, alternative direction method with multipliers, block coordinate descent methods and augmented Lagrange methods [13], [50], [103]. Furthermore, with non-linear functions, other regression-based methods like tree-based method [97] are also applied to fit expression data.

#### 2.2.1 GENIE3

Gene network inference with ensemble of trees (GENIE3) is a tree-based method to reconstruct GRNs [97]. Although it was originally designed for bulk transcriptomes, it has also been used in scRNA-seq data [83] because of its good performance in GRN reconstruction from bulk transcriptomes [68]. The input expression data is an N×G matrix, where the expression of G genes are quantified in N experiments (or cells). GENIE3 assumes that the expression of each gene could be described as a function of the expression of some TFs, which means the selected TFs could regulate the target gene. Thus, the inference of GRNs is decomposed into G different regression problem for all target genes.

Denote the expression of gene j and all genes except gene j in the kth experiment (or cell) by $x_{j,k}$ and $x_{-j,k}$, respectively. The major objective of GENIE3 is to find a suitable function $f_{j}$ for gene j such that

$$x_{j,k}=f_{j}\left(x_{-j,k}\right)+\varepsilon_{k},\forall k\in \left[1,N\right],$$

where $\varepsilon_{k}$ represents a random noise with zero mean. Regression tree [15] is a good candidate to seek such function and identify those TFs that could be used to predict the expression of gene j. Based on regression tree, random forests [14] is able to reduce the variance and improve the performance [42]. In addition, random forests is able to avoid the overfitting phenomenon and requires little tuning parameters. Consequently, random forests is applied in GENIE3 for each gene to identify the TFs used to predict.

In random forests, m variables (e.g., TFs) are randomly selected from G variables as split candidates at each node, and K single regression trees are built by K bootstrapping. Importance measure (IM) is defined to quantify how relevant each TF (input gene) is to the target gene (output gene) and is computed for each single regression tree. The attribute IM is extended by averaging the IMs over K regression trees in random forests; see section Methods in [97] for details. By ranking G IMs from every single ensembled tree and aggregating them to get global interaction ranking, the final GRN is inferred by setting a threshold to define the regulatory links.

Benefitting from the fact that few assumptions are required in random forests, GENIE3 owns ability to explain more complex regulatory relationships in GRNs when comparing with linear regression. GENIE3 is a good choice for scRNA-seq data without temporal information, while it might perform worse than other methods if scRNA-seq data contains temporal information. In addition, it may be harder for GENIE3 to infer large networks when it is needed to build G×K regression trees one by one, while the computational difficulty can be relieved by parallel computation. For example, a large network (e.g., with 5000 genes) could still be inferred in practice [83].

#### 2.2.2 SINCERITIES

Single-cell regularized inference using timestamped expression profiles (SINCERITIES) applies regularized linear regression and partial correlation analysis to reconstruct GRNs based on temporal changes in the distributions of gene expression [80]. This method assumes the expression change of a target gene linearly depends on the expression changes of TFs at a time delay.

Such temporal changes in the expression of each gene is measured by the distance of gene expression distributions between two subsequent time points, which is called as the distributional distance (DD). Kolmogorov-Smirnov distance is used to compute the DDs of all genes [69] and ${\hat{\mathrm{DD}}}_{j,l}$ denotes the normalized DD of gene j at time window l. Based on the assumption mentioned above, SINCERITIES reconstructs GRNs by solving G linear regressions for G genes. More precisely, the linear regression for target gene j at time window l+1 is formulated as:

$${\hat{\mathrm{DD}}}_{j,l+1}=A_{1,j}{\hat{\mathrm{DD}}}_{1,l}+A_{2,j}{\hat{\mathrm{DD}}}_{2,l}+\cdots +A_{G,j}{\hat{\mathrm{DD}}}_{G,l},$$

where $A_{j}:={\left[A_{1,j},A_{2,j},\cdots ,A_{G,j}\right]}^{⊤}$ represents the coefficients in linear regression. Since the number of genes is larger than the number of time windows in general, SINCERITIES applies an $l_{2}$ norm penalized linear regression (ridge regression) [46] to overcome the difficulty of solving the underdetermined equations for target gene j, that is,

$$\min_{A_{j}}{\|Y_{j}-XA_{j}\|}^{2}+\lambda {\|A_{j}\|}^{2},$$

where

$Y_{j}:={\left[{\hat{\mathrm{DD}}}_{j,2},{\hat{\mathrm{DD}}}_{j,3},\cdots ,{\hat{\mathrm{DD}}}_{j,n-1}\right]}^{⊤}$ and $X:=\left[\begin{array}{ccc} {\hat{\mathrm{DD}}}_{1,1} & {\hat{\mathrm{DD}}}_{2,1} & \begin{array}{cc} \cdots & {\hat{\mathrm{DD}}}_{G,1} \end{array}\\ {\hat{\mathrm{DD}}}_{1,2} & {\hat{\mathrm{DD}}}_{2,2} & \begin{array}{cc} \cdots & {\hat{\mathrm{DD}}}_{G,2} \end{array}\\ \begin{array}{c} \vdots \\ {\hat{\mathrm{DD}}}_{1,n-2} \end{array} & \begin{array}{c} \vdots \\ {\hat{\mathrm{DD}}}_{2,n-2} \end{array} & \begin{array}{cc} \cdots & \vdots \\ \cdots & {\hat{\mathrm{DD}}}_{G,n-2} \end{array} \end{array}\right].$

After ranking the absolute values of the coefficients of all possible edges, the inferred GRN could be obtained by setting a threshold for the ranked value. The sign of the regulatory edge between each pair of TF and target is determined by the sign of the corresponding partial correlation.

SINCERITIES reconstructs the GRNs with low computational complexity and suits for high-dimensional data (e.g., a network with 5000 genes) [80], [83]. As the regressions for all genes are independent of each other, the running time could be depleted by employing parallel computation technique. However, temporal information is required in this method, and the relationship between temporal changes in the expression of TFs and target gene may not be linear as assumed to be.

### 2.3 Correlation/information-based model

The regulatory links in GRNs can also be determined by measuring the relationship between the expression of target genes and TFs. The Pearson's correlation, is the simplest statistic to characterize the association between X and Y:

$$\rho_{X,Y}:=\frac{cov(X,Y)}{\sigma_{X}\sigma_{Y}}=\frac{E[\left(X-\mu_{X}\right)\left(Y-\mu_{Y}\right)]}{\sigma_{X}\sigma_{Y}},$$

where $\mu_{X}$ and $\sigma_{X}$ denote the mean and variance of variable X, respectively, cov(X,Y) represents the covariance between X and Y, and $E\left(\cdot \right)$ denotes the expectation.

However, the Pearson's correlation is too naive to characterize the complicated regulatory relationship in GRNs. For example, if genes i and j are not connected but both connected to gene k, the correlation between i and j is still possible to be high. Partial correlation [58] could be used to avoid the effect of other genes. It can be quickly obtained by computing the correlation between the residuals from two corresponding linear regressions, which means that the linear relationship is assumed.

In information theory, the entropy $H\left(X\right)$ is used to measure the uncertainty of random variable X. If the random variable Y is known, one may define another concept called conditional entropy $H\left(X|Y\right)$ [24]. These two basic concepts are defined as

$$H\left(X\right):=-\sum_{x\in X}p\left(x\right)\log p\left(x\right)$$

and

$$H\left(X|Y\right):=-\sum_{x\in X,y\in Y}p\left(x,y\right)\log\frac{p\left(x,y\right)}{p\left(y\right)},$$

respectively.

By considering the distributions of genes, mutual information (MI) has the ability to quantify the dependence between two genes based on their distributions. MI for two random variables X and Y is formulated as

$$I\left(X;Y\right):=\sum_{x\in X}\sum_{y\in Y}p\left(x,y\right)\log\left(\frac{p\left(x,y\right)}{p\left(x\right)p\left(y\right)}\right)=H\left(X\right)-H\left(X|Y\right).$$

The second equality shows the relationship between MI and entropy. From the formula mentioned above, MI measures the reduction in uncertainty of a random variable X when the knowledge of variable Y is known. Considering the effect from a third variable Z, conditional MI is used to measure the reduction in the uncertainty of X due to knowledge of Y when Z is given [24], which is formulated as

$$I\left(X;Y|Z\right):=\sum_{z\in Z}p\left(z\right)\sum_{x\in X}\sum_{y\in Y}p\left(x,y|z\right)\log\left(\frac{p\left(x,y|z\right)}{p\left(x|z\right)p\left(y|z\right)}\right).$$

However, the estimation of MI and conditional MI involves data discretization and estimation of empirical probability distributions [18], and thus different choices of discretization method and estimator for MI would affect the performance of MI-based method [26], [98], [109].

The inferred regulatory link is more reliable when the value of measurements is larger. After computing these measurements mentioned above for all genes, those links with lower values could be removed by choosing a threshold to infer the final GRNs.

#### 2.3.1 LEAP

Lag-based expression association for pseudo-time series (LEAP) is a correlation-based algorithm to infer the GRNs for pseudo-time-series data [90]. As LEAP is developed based on the Pearson's correlation, the linear relationship between a pair of genes is always assumed [61].

Given expression data $x_{i,t}$ of gene i at time t∈{1,2,⋯,T}, the series $X_{i,l}:=\left\{x_{i,l+1},x_{i,l+2},\cdots ,x_{i,l+s}\right\}$ for gene i is extracted by setting windows of size s, where the lag l∈{0,1,⋯,T-s}. Instead of Pearson's correlation, LEAP uses maximum absolute correlation (MAC) to measure the regulatory relationship:

$$\rho_{\mathrm{ij}}^{*}:=\max_{l\in \{0,1,\cdots ,T-s\}}\left|\rho_{\mathrm{ijl}}\right|,$$

where $\rho_{\mathrm{ijl}}$ denotes the Pearson's correlation between gene i at lag 0 ($X_{i,0}$) and gene j at lag l ($X_{i,l}$). The directional regulatory relationship could be inferred by the value $l^{*}=\arg\max_{l\in \{0,1,\cdots ,T-s\}}\left|\rho_{\mathrm{ijl}}\right|$ and the corresponding MAC value $\rho_{\mathrm{ij}}^{*}$: (1) if $l^{*}\neq 0$, the MAC value $\rho_{\mathrm{ij}}^{*}>0$ and $\rho_{\mathrm{ij}}^{*}<0$ represents that the gene i activates and inhibits gene j, respectively; (2) if $l^{*}=0,$ gene i, and j are both regulated by a third gene. Finally, the statistical significance can be calculated based on the false discovery rate [8].

The LEAP provides a strategy to find the regulatory links between genes and define their directional relationship by computed measurements. However, the relationships between all genes are assumed to be linear, where it might not satisfy for most cases. As the temporal information is considered in the method, pseudo-time-series data is required to infer GRNs. In practice, this correlation-based model generally consumes less time because the measurements can be directly computed by the analytical formulas, and it works for a large network. For example, a network with 5000 genes is considered in [83].

#### 2.3.2 PIDC

Partial information decomposition and context (PIDC) is an information-based algorithm to infer the regulatory relationship between genes [18]. Partial information decomposition (PID) is used to decompose the multivariate MI, where unique information ${Unique}_{Z}\left(X;Y\right)$ is the portion of information provided only by Y [100]. To quantify the information between multiple genes in GRNs, PIDC defines a new measurement called proportional unique contribution (PUC) between genes X and Y, which is the sum of the ratio ${Unique}_{Z}\left(X;Y\right)/I\left(X;Y\right)$ for all other genes Z in set S. The ratio eliminates the impact from the quantity of MI, and the computation of PUC could be formulated as

$$u_{X,Y}:=\underset{Z\in S_{\backslash \left\{X,Y\right\}}}{\sum }\frac{{\mathrm{Unique}}_{Z}\left(X;Y\right)}{I\left(X;Y\right)}+\underset{Z\in S_{\backslash \left\{X,Y\right\}}}{\sum }\frac{{\mathrm{Unique}}_{Z}\left(Y;X\right)}{I\left(X;Y\right)}.$$

A global threshold for PUC scores might bias the result of the inferred GRNs due to the distributions of PUC scores differ between genes [18]. The confidence of a regulatory link between a pair of genes could be calculated by the empirical probability distribution estimated from PUC scores; see section Results in [18] for details.

The PIDC provides an approach to quantify the relationship between a pair of genes considering the effect of other related genes in GRNs. It extracts more information from the expression data. However, the data discretization and MI estimators are required in this method, which might impede the computation of PUC scores. The performance of PIDC might be influenced by the choice of data discretization methods and MI estimators [18]. Although the method owns high complexity, the problem could be relieved by implementing in Julia programming language to speed up [10]. Moreover, it is capable of dealing with a large network (e.g., with 5000 genes) in practice [83].

#### 2.3.3 Scribe

Scribe is another information-based toolkit designed for datasets with temporal information to infer causality relationship between genes. It relies on restricted directed information (RDI) [87] to measure the information transmitted from potential regulators to downstream targets. The GRNs can be correctly reconstructed based on the assumption that the underlying processes can be described by a first-order Markov process, which is true in most biological processes [87]. To measure information transferred from the regulator X at time t-d to Y at time t with time delay d when the information of Y at time t-1 is given, the computation of RDI is formulated in the form of conditional MI:

$${RDI}_{d}\left(X\to Y\right):=I\left(X_{t-d};Y_{t}|Y_{t-1}\right).$$

Furthermore, conditional RDI (cRDI) is considered to remove the arbitrary effect from other potential regulators Z, and thus the computation of cRDI can be formulated as:

$${RDI}_{d_{1}}\left(X\to Y|Z_{t-d_{2}}\right):=I\left(X_{t-d_{1}};Y_{t}|Y_{t-1},Z_{t-d_{2}}\right).$$

To correct the sampling bias in computation and improve the performance, uniformized RDI and cRDI scores are computed by replacing the original empirical distribution of the samples with a uniform distribution [86]. The final GRN is generated by the RDI-based scores and further refined by context likelihood of relatedness algorithm [33] with graph regularization method; see section STAR Methods in [86] for details.

Scribe extracts more intrinsic information from single-cell expression data by considering arbitrary effect delay from regulator X to target Y and the effect from other potential regulators Z. It quantifies the regulatory causality between X and Y based on the time information. Also, Scribe can detect both linear and non-linear causality in GRNs [86]. However, as the RDI is one type of conditional MI, the Scribe involves the estimation of RDI-based measurements, which might be time-consuming. In practice, a network with 1000 genes can be reconstructed by this method [83]. In addition, scRNA-seq data with temporal information is required because the time information is needed. As several methods mentioned above, Scribe can analyze pseudo-time series and RNA velocity.

### 2.4 Boolean network

Unlike the continuous expression values of the nodes in ODE, Boolean network describes the interaction of genes with discrete values for their states along with discrete time points. The nodes and edges of the network represent genes and regulatory relationships between them, respectively. To represent the expression status of genes, the numeric "1" or "0" is used to denote the state of nodes as "on" or "off". In order to characterize the dynamics of the network, Boolean functions with three main operations: AND, OR and NOT are built to update the successive state for each node, where the operators represent the regulatory manners of TFs to their targets. The final successful model can be obtained by verifying the dynamic sequence of system states and comparing with biological evidence. A drawback of Boolean network is that the computation consumes more time when more possible networks are needed to be considered with an increasing number of genes. Thus, the method is limited in a small number of genes in real practice (generally smaller than 100) [35], [65]. The method would be sensitive to dropouts since the binarization of expression data is required before modeling [35], [106]. The example showed below simply illustrates the Boolean network for three nodes.

**Fig. 3:**
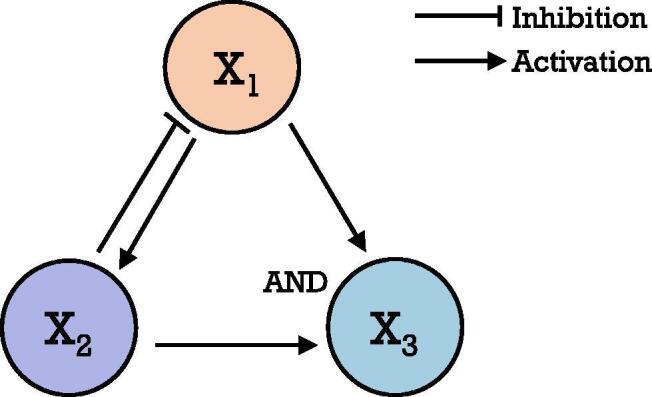
Three nodes network.

**Example 1.** Consider the following network with three nodes as $X_{1}$, $X_{2}$ and $X_{3}$ (the Fig. 3 there)

The Boolean update functions can be presented as follow:

$$\begin{array}{c} X_{1}\left(t+1\right)=\mathrm{NOT}\;X_{2}\left(t\right);\\ X_{2}\left(t+1\right)=X_{1}\left(t\right);\\ X_{3}\left(t+1\right)=X_{1}\left(t\right)\mathrm{AND}\;X_{2}\left(t\right), \end{array}$$

where $X_{1}\left(t\right)$ denotes the state of the node $X_{1}$ at the time t.

#### 2.4.1 SCNS toolkit

Single cell network synthesis toolkit (SCNS toolkit) is a Boolean network-based toolkit for scRNA-seq data with temporal information to reconstruct and analyze GRNs. The diffusion map method [23] is used to identify the developmental trajectories in gene expression data from different cell stages [74].

The SCNS toolkit firstly discretizes the single-cell gene expression into binary states, where "1" and "0" represent that a gene is expressed or not respectively. According to the Boolean update functions that represent connections of a possible network, the vector bearing "1" or "0" states of all genes at an early time point can transit into the state vector of the next time point. State vectors at two adjacent time points could be connected to form a state transition graph. Boolean functions that fit the state series best are being chosen when the network is being reconstructed; see section Implementation in [102] for details.

The SCNS toolkit provides insights into the developmental processes and the interactions between genes in GRNs across time. It considers regulatory logic when reconstructing the GRNs. Yet the method for data discretization in SCNS toolkit might influence the further inference of GRNs. As we mentioned above, the Boolean network-based method can only deal with the small-scale GRNs in real-life computation.

## 3 Methods for scRNA-seq data with genome

Although scRNA-seq data are widely used for GRN reconstruction, the performance of current tools on this data type is still unsatisfactory [20], [83]. This is because, with similarity to those designed for bulk RNA-seq, these tools are all based on the assumption that the expression relationships between a target gene and its TFs imply transcriptional regulations among them. However, the observed associations between TFs and genes may be due to other biological events or even randomness rather than transcriptional regulations. Given the stochastic variation of gene expression in single cells, the dropouts and technical variations of scRNA-seq data, the signal-to-noise ratio of scRNA-seq is even lower than that of bulk RNA-seq. Besides, based on scRNA-seq data alone, it is also difficult to distinguish between direct and indirect regulations. To overcome these issues and improve the performance of GRN inference, integration of additional data is considered as an improved way. Genome sequences bearing the genomic regulatory codes can be exploited to guide the identification of potential TF binding. A TF binding motif located at the DNA regulatory element of a gene indicates a potential direct regulation between them.

Single-cell regulatory network inference and clustering (SCENIC) is one of such tools [2]. It incorporates the promoter sequences extracted from the reference genome to search direct connection between TFs and their target among the coexpression network modules built by GENIE3 [97] or GRNBoost [2]. By removing the indirect targets lacking enriched motif detected using RcisTarget [2], SCENIC dramatically reduces the false connections in the GRN inferred from scRNA-seq alone [2]. It also quantifies the subnetwork activity in each cell by the AUCell algorithm [2], which allows the comparison of the activities of cell-specific networks among different cell types and subpopulations. It enables the combination of coexpression networks with cis-regulatory analysis, leading to a better exploration of GRNs and cell states. Thus, the datasets with complex cell states can also achieve good performances. When dealing with very large datasets, GRNBoost, a variant of GENIE3, can advance the efficiency and reduces the time used in GRN reconstructions. The SCENIC provides a strategy to discover interactions between TFs and target genes, yet the inference of the coexpression network might affect the further analysis. The SCENIC might perform better with other methods when it is inferring coexpression networks.

However, when the majority of associated genetic variants locates in regulatory regions of patient genomes in diseases like cancer [73], the reference genome is unable to reflect the heterogeneity of regulatory codes in cell populations. Regulatory variants in different cell subpopulations may drive the regulations on diverse patterns of gene expression. Thus, integration of scRNA-seq and single-cell genome sequencing will be a better strategy to understand the heterogeneity of GRNs in a tumor cell population. Although technologies, such as G&T-seq [67] and DR-seq [27], allow parallel sequencing of the genome and transcriptome in the same single cell, the high cost of sequencing covering the whole genomes for thousands of single cells and relatively low resolution of the technique have limited the popularization of this approach. Thus, so far, no bioinformatics tools were especially designed for this analysis. However, it is still worthy to develop such tool especially for cancer research, when targeted genome sequencing may dramatically reduce the sequencing cost by selecting genes and genomic regions of interests [75].

## 4 Methods for scRNA-seq data with single-cell epigenomes

Fortunately, the development of single-cell epigenomic technologies, such as scATAC-seq, allows the identification of DNA regulatory elements in single cells at a reasonable cost. Open chromatin regions detected by scATAC-seq often contain active DNA regulatory elements for TF binding and gene regulations [16]. Thus, scATAC-seq is able to identify direct regulations in GRNs. The integration of bulk RNA-seq and bulk ATAC-seq (or other epigenomic data) has been proved to improve the accuracy of GRN inference significantly [1], [84], [99]. This approach is also applicable to single-cell sequencing data. However, due to cell-type/condition specificity of transcriptome and epigenome profiles, the integration of bulk RNA-seq with bulk ATAC-seq/ChIP-seq usually requires that the two data sets are derived from the same cell type and in the same condition. Although several technologies allow sequencing transcriptome and epigenome simultaneously in the same cell [4], [19], [49], researchers often conduct scRNA-seq and single-cell epigenome separately, so the major challenge for the integration approach is how to match the cell clusters of the same cell type, condition or cell state for the two sequencing data types respectively. Since scATAC-seq is more commonly used for single-cell epigenome profiling than other techniques like scChIC-seq, three bioinformatics tools have been introduced to combine scRNA-seq and scATAC-seq data for GRN reconstruction. These methods can analyze more than ten thousand genes, and they are applicable to high-dimensional matrices during multi-omics data integration (Table 1).

### 4.1 SOM

Self-organizing map (SOM), also known as the Kohonen network, is an unsupervised learning method for clustering and visualization [55], [56]. The main structure of SOM is separated into two parts: an input layer and a competitive layer (also as output layer). The competitive layer is generally a two-dimensional array of output nodes that are assumed to be a regular hexagonal or rectangular grid.

Denote n nodes in input layer by

$$X:=\left[x_{1};x_{2};\cdots ;x_{m}\right]\in R^{m\times n},$$

where $x_{u}\in R^{n}$ is the uth input vector (e.g. the uth sample in expression data). Each unit i in competitive layer is connected to input layer by a weight vector

$$w_{i}:={\left[w_{i1},w_{i2},\cdots ,w_{\mathrm{in}}\right]}^{⊤}\in R^{n},$$

where $w_{\mathrm{ij}}$ denotes the weight for the connection between unit i and node j (e.g., gene j) in input layer. The iterative computation in SOM involves searching a winning unit k in competitive layer based on the minimal Euclidean distance

$$k=\underset{i}{\arg\min}{\left\|w_{i}-x_{u}\right\|}^{2}$$

or the maximal inner product

$$k=\underset{i}{\arg\max}\;w_{i}^{⊤}x_{u}.$$

Given a random initial weight vector $w_{i}\left(0\right)$ for each unit i, the weights for the neighborhood of winning unit k are updated by

$$w_{i}\left(l+1\right)=w_{i}\left(l\right)+\eta \left(l\right)h_{\mathrm{ki}}\left(l\right)\left[x_{u}-w_{i}\left(l\right)\right],\forall i\in O_{k},$$

with a learning rate $\eta \left(l\right)$, where $O_{k}$ denotes a set of unit k's neighborhood (based on the structure in the competitive layer), and $h_{\mathrm{ki}}\left(l\right)$ is the neighborhood function for unit k; see [56] for more details.

The SOM has the ability to map data from a high dimension space to a low dimension one. Although the convergence of the algorithm has been proved under some conditions, the SOM might converge until hundreds of thousands of iterations [11]. Thus, SOM is computationally expensive compared with other clustering methods.

#### 4.1.1 LinkedSOMs

Linked self-organizing maps (LinkedSOMs) is a bioinformatics tool developed to infer GRNs by integrating scRNA-seq and scATAC-seq data. The input data for LinkedSOMs are the gene expression data and chromatin data, while the pseudo-time is not required. Two SOMs with the output set of SOM units are available after training the scRNA-seq and scATAC-seq data separately. K-means clustering [36] is then performed to determine centroids among units, and the cluster of the units, called metaclusters, are built around these centroids based on Akaike information criterion score [3]. To link gene expression and chromatin accessibility, GREAT algorithm [71] is implemented to obtain the linked SOM metaclusters (LMs). The underlying GRNs are then inferred after gene ontology analysis and motif analysis on these LMs; see section Methods in [52] for details.

Training two SOMs for scRNA-seq and scATAC-seq datasets makes LinkedSOMs time-consuming as mentioned above, though it can still analyze large datasets. Even though the original study of LinkedSOMs focuses on integrating scRNA-seq and scATAC-seq data, it is also applicable to multi-omics data analysis incorporating other single-cell sequencing data.

### 4.2 NMF

Nonnegative matrix factorizations (NMF) aims to decompose a nonnegative matrix $X{\in R}^{n\times m}$ into two nonnegative matrices $W{\in R}^{n\times r}$ and $H{\in R}^{r\times m}$ such that X≈WH [59]. The approach to find W and H is by solving the minimization problem

$$\underset{W,H\geq 0}{min}{\left\|X-WH\right\|}_{F}^{2},$$

where ${\left\|\cdot \right\|}_{F}$ denotes the Frobenius norm. Via the NMF, the matrix X could be approximately represented as linear combinations of r column vectors in feature matrix W with assignment weight matrix H. The NMF method has been widely applied to GRN inference [77], [105], [108]. Many methods are developed to solve the NMF problem, such as simple multiplicative update method [60] and projected gradient method [66]. To the best of our knowledge, the convergence properties of the projected gradient method have been proved, while the convergence properties of simple multiplicative update method are still not clear [66], [92].

#### 4.2.1 Coupled NMF

Coupled nonnegative matrix factorizations (coupled NMF) is an NMF-based approach to reconstruct GRNs via integrative analysis of scRNA-seq and scATAC-seq data. The main assumption in coupled NMF is that the expression of a subset of genes (detected by scRNA-seq) can be linearly predicted from the status of chromatin regions (detected by scATAC-seq).

Coupled NMF aims to cluster the cells in each dataset with information from another one by developing a new optimization problem based on NMF. Denote the scRNA-seq and scATAC-seq data by X and O, respectively. Borrowing the idea from NMF and introducing the coupling matrix A to connect the clusters $W_{1}$ and $W_{2}$ of two datasets, the coupled NMF is formulated as

$$\underset{W_{1},H_{1},W_{2},H_{2}\geq 0}{\min}\frac{1}{2}{\|O-W_{1}H_{1}\|}_{F}^{2}+\frac{\delta_{1}}{2}{\|X-W_{2}H_{2}\|}_{F}^{2}-\delta_{2}tr\left(W_{2}^{⊤}AW_{1}\right)+\delta_{3}\left({||W_{1}||}_{F}^{2}+{||W_{2}||}_{F}^{2}\right),$$

where $\delta_{k}\left(k=1,2,3\right)$ are the penalty parameters in this optimization problem. The trace term $tr\left(W_{2}^{⊤}AW_{1}\right)$ owns ability to induce the consistency of features $W_{2}$ with linear transformed features $AW_{1}$. The last term in objective function controls the growth of $W_{1}$ and $W_{2}$ [30]. Before solving the coupled NMF mentioned above, the coupling matrix A is firstly obtained by performing the regression model on the paired gene expression and chromatin accessibility data. The coupled NMF is then solved by a modified multiplicative update algorithm [30]. The method finally generates the cluster-specific expression of genes and accessibilities of regulatory elements, where the cluster-specific expression of genes can be predicted from the cluster-specific accessibilities of regulatory elements by $AW_{1}$. After gene ontology analysis and motif analysis on each cluster, in the end the final GRNs can be reconstructed, see section Materials and Methods in [30] for details.

Similar to LinkedSOMs discussed above, other single-cell multi-omics data can also be applied in this approach to analyze and infer the GRNs with coupled NMF. Although the numerical behavior of coupled NMF was showed [30], the convergence properties have not been established yet.

### 4.3 CCA

Canonical correlation analysis (CCA) is a method to project two different datasets into a correlated low-dimensional space by maximizing the correlation between two linear combinations of the features in each dataset [47]. Denote two datasets by X and O. Introducing the linear combinations as U:=Xu and V:=Ov with two canonical correlation vectors (CCVs) u and v, the CCA can be described as pursuing the maximum correlation of linear combinations U and V:

$$\underset{u,v}{max}\;corr\left(U,V\right).$$

Supposed that the columns of X and O have been centered and scaled, the problem can be re-written as

$$\begin{array}{ll} \underset{u,v}{\max} & u^{⊤}X^{⊤}Ov\\ \text{s.t.} & u^{⊤}X^{⊤}Xu\leq 1,\\ & v^{⊤}O^{⊤}Ov\leq 1. \end{array}$$

The solution (u and v) of CCA can be obtained by solving a standard eigenvalue problem [47], [96]. When it comes to high-dimensional application, its performance achieves a better result if it treats the covariance matrices of X and O as diagonal matrices [29], [95]. By replacing the $X^{⊤}X$ and $O^{⊤}O$ with the identity matrices, the modified optimization problem called diagonal CCA is reformulated as

$$\begin{array}{cc} \underset{u,v}{\max} & u^{⊤}X^{⊤}Ov\\ \text{s.t.} & \|u{\|}^{2}\leq 1,\\ & \|v{\|}^{2}\leq 1, \end{array}$$

and it can be solved by penalized matrix decomposition [101].

#### 4.3.1 Seurat v3

Seurat v3 is a bioinformatics framework that can infer GRNs from scRNA-seq and scATAC-seq data based on CCA. Denote the scRNA-seq and scATAC-seq data by X and O, respectively. The CCVs u and v are generated by performing diagonalized CCA with standard singular value decomposition method, which is followed by $l_{2}$-normalization on CCVs to eliminate global differences in scale across datasets. For each cell in one dataset, its K-nearest neighbors (KNNs) in another dataset can be identified in the shared low-dimensional space based on the $l_{2}$-normalized CCV. If a pair of cells from each dataset is contained in each other's KNN, the pair of cells is defined as the mutual nearest neighbor (MNN), also called anchor [40], [91]. Then the anchors are scored and filtered to alleviate the effects of any incorrectly identified anchors. After converting scATAC-seq data into a predicted gene expression matrix [81], an integrated expression matrix for scRNA-seq and scATAC-seq is finally computed with the strategy in batch correction [40]. The GRNs can be inferred with this expression matrix as input via any single-cell GRN inference method; see section Method Details in [91].

The Seurat v3 focuses on the integration of scRNA-seq with different single-cell technologies including scATAC-seq. It generates an integrated expression matrix in the end, which can be the input in further downstream analysis like GRN inference with any single-cell analytic method. Moreover, the approach in Seurat v3 is extended to assemble multiple datasets, and this would provide a deeper insight into single cells. In addition, based on the principle of CCA and KNN, the Seurat v3 is capable of dealing with high-dimensional datasets.

## 5 Conclusions

With the development of various single-cell sequencing technologies nowadays, more and more methods for GRNs inference from single-cell sequencing data are proposed [12], [20], [31], [35], [83]. Understanding the mathematical background of each method might help researchers use these methods appropriately in different cases. It also benefits the tool developer to design new tools with comprehensive considerations. This review introduces various single-cell sequencing data available for GRN reconstruction. Then mathematical principles and adaptabilities of several popular algorithms that have been applied to scRNA-seq data alone or integrative multiple single-cell data are discussed. For each representative tool, the acceptable data type and underlying assumption are emphasized to point out the specific circumstance where the method could be applied.

As the proverb says, "Essentially, all models are wrong, but some models are useful". Although comparisons on several tools that work on scRNA-seq data have been performed with simulated data and real data in several published reviews [20], [83], it is still difficult to conclude which method is the best. First, in general, it seems that there is no method that significantly outperforms others in all datasets, especially on real datasets. Second, since GRNs are highly condition-specific and largely unknown, the GRNs inferred by these tools from real scRNA-seq data are hard to be well evaluated. Current comparisons on their performance are usually based on "gold standard" of non-specific networks or very limited known network connections under the benchmarking data. While methods for integrative multiple single-cell data have the same issues. Thus, we only discuss their adaptabilities and limitations based on their basic algorithm here. Further comparison on the accuracy of GRNs that they predict from real data requires more good benchmarking data and corresponding verified gold standard networks, which is not available now.

We also point out that the future direction of method development would be the integration of multiple single-cell sequencing data. Integrations of single-cell multi-omics could reduce the impacts of noise and enhance the performance by cross-validating the regulatory connections in GRNs through multiple datasets. More integrative tools will emerge when more types of single-cell data, such as proteome, metabolome, cell image, et al., become prevalent in the future. They will depict gene regulatory mechanisms underlying disease and biological processes more accurately, and provide a more comprehensive map of GRNs covering multiple biological molecules and regulatory layers. In addition to the integration of multiple data types, combining multiple algorithms and tools has also been shown to improve the accuracy of network inference from bulk-cell data [68]. We speculate that the same phenomenon will occur for single-cell data. Thus, new tools considering multiple algorithms may further improve the prediction of GRNs from single-cell sequencing data.

## Conflict of interest

We have no conflict of interest.

## CRediT authorship contribution statement

**Xinlin Hu:** Writing - original draft, Data curation, Visualization. **Yaohua Hu:** Conceptualization, Writing - review & editing, Supervision, Funding acquisition. **Fanjie Wu:** Data curation, Visualization. **Ricky Wai Tak Leung:** Writing - review & editing, Visualization. **Jing Qin:** Conceptualization, Writing - original draft, Supervision, Project administration.
